# Two TPX2-Dependent Switches Control the Activity of Aurora A

**DOI:** 10.1371/journal.pone.0016757

**Published:** 2011-02-09

**Authors:** Xue Xu, Xia Wang, Zhengtao Xiao, Yan Li, Yonghua Wang

**Affiliations:** 1 Center of Bioinformatics, Northwest A&F University, Yangling, China; 2 School of Chemical Engineering, Dalian University of Technology, Dalian, China; 3 Lab of Pharmaceutical Resource Discovery, Dalian Institute of Chemical Physics, Chinese Academy of Sciences, Dalian, China; University of South Florida College of Medicine, United States of America

## Abstract

Aurora A is an important oncogenic kinase for mitotic spindle assembly and a potentially attractive target for human cancers. Its activation could be regulated by ATP cycle and its activator TPX2. To understand the activation mechanism of Aurora A, a series of 20 ns molecular dynamics (MD) simulations were performed on both the wild-type kinase and its mutants. Analyzing the three dynamic trajectories (Aurora A-ATP, Aurora A-ADP, and Aurora A-ADP-TPX2) at the residue level, for the first time we find two TPX2-dependent switches, i.e., switch-1 (Lys-143) and switch-2 (Arg-180), which are tightly associated with Aurora A activation. In the absence of TPX2, Lys-143 exhibits a “closed” state, and becomes hydrogen-bonded to ADP. Once TPX2 binding occurs, switch-1 is forced to “open” the binding site, thus pulling ADP away from Aurora A. Without facilitation of TPX2, switch-2 exits in an “open” conformation which accompanies the outward-flipping movement of P·Thr288 (in an inactive conformation), leading to the crucial phosphothreonine exposed and accessible for deactivation. However, with the binding of TPX2, switch-2 is forced to undergo a “closed” movement, thus capturing P·Thr288 into a buried position and locking its active conformation. Analysis of two Aurora A (K143A and R180A) mutants for the two switches further verifies their functionality and reliability in controlling Aurora activity. Our systems therefore suggest two switches determining Aurora A activation, which are important for the development of aurora kinase inhibitors.

## Introduction

Aurora-A, as a member of the Aurora family of serine-threonine kinases, localizes to centrosomes and proximal mitotic spindles and regulates spindle-associated events during early mitosis [1]. It functions mainly in centrosome separation and maturation, spindle assembly and stability, chromosome condensation, and cytokinesis in mammalian cells [2], [3]. This kinase is overexpressed in a wide range of tumor types at high frequency compared with essentially nonproliferating matched normal tissue, which is associated with amplification of the region of chromosome 20 encoding AURKA [4]. Increased Aurora A expression cause increased kinase activity, finally contributing to tumor initiation and progression [5]. This oncogenic role of Aurora A, coupled to its essential role in mitotic progression, thus make inhibition of Aurora's activity become a prominent strategy in the development of rational cancer therapeutics [6].

The kinase activity of Aurora A is tightly regulated by the ATP cycle in the ATP binding site [7] that is located at the interface of catalytic core, comprised of an N-terminal lobe (residues 123–210) and a large C-terminal lobe (residues 217–387). This region involves a complex kinetic cycle of ATP binding, nucleotide hydrolysis, and sequential release of the products Pi and ADP, respectively [8]. A steady cycle of ATP is so critical that an inhibitor which blocks certain step of this cycle deactivates the kinase in seconds [9]. During the process of ATP hydrolysis, conformational coupling to this step allows functionally important conformational transitions of a contiguous segment of the C-terminal domain known as the “activation segment” (residues 274–299) (see Figure S1). The conformational change within the activation segment involves phosphorylation of a conserved threonine residue (Thr-295 in Xenopus, Thr-288 in human) [7] critical for the activity of Aurora A, although it is unclear as to the regulation mechanism of phosphorylation for the kinase activity. This autocatalytic activity of Aurora-A is facilitated by several partner proteins, such as the microtubule-associated protein TPX2, Ajuba [10], protein phosphatase inhibitor-2 [11], the focal scaffolding protein HEF1[12], and a newly identified Bora [13]. TPX2 is one of the most efficiently phosphorylated Aurora A partner proteins. Upon TPX2 binding, the in vitro autophosphorylation activity of Aurora A is increased, and dephosphorylation is prevented [14], [15]. The coprecipitation assay for the interaction of PP1 (protein phosphatase 1) and Aurora A using a phosphospecific P·Thr-288 antibody shows the band corresponding to protected P·Thr-288 is only found in the present of full length human TPX2 or TPX2 1–43, which indicates that TPX2 definitely protects residue Thr-288 from dephosphorylation. The TPX2 fragment binds Aurora-A with two separate stretches [16]. The upstream stretch (residues 7–21) binds to the N-terminal lobe of Aurora A in a mostly extended conformation. The downstream stretch (residues 26–43) adopts an α-helical conformation that interacts with both the helix aC and the activation segment of Aurora A.

Although the crystal structures of phosphorylated Aurora-A have been unveiled [16], [17], several fundamental questions related to the regulation of Aurora A activity by TPX2 remain unclear. An unsolved question is how TPX2 binding is linked to efficient ATP cycle in Aurora, and how this cycle coupled with the binding of TPX2 affect the kinase activity.

However, experimental evidence for the dynamics of ATP-, ADP- and TPX2-bond Aurora A is very hard to obtain. Much of the experimental information about protein dynamics has come from superposable crystal structures [15]. Therefore, molecular dynamics (MD) simulations, as a powerful method to study the mobility of proteins, is applied in this work to investigate the mechanism of activation modulation of the Aurora A conformational dynamics at atomic resolution.

## Results and Discussion

### 1 Switch-1 in the ATP binding site

Since the kinetic cycle of ATP is the essential step to make the kinase catalytically active [17], inhibition of the ATP cycle can induce a deactivation process of Aurora A, which makes the ATP binding site of Aurora-A a novel target for cancer therapy [18]. For Aurora-A, ATP is captured in the ATP binding site sandwiched between the C- and N-termini. Upon ATP hydrolysis, a conformational transition of the binding site is triggered by the binding of TPX2, which facilitates the transfer of phosphate group and the release of ADP [19]. Although structural and mutational studies [15], [18] have identified several residues involved in Aurora A activity, it is still unclear which residues do play roles in the regulation of the ATP cycle, how the residues contact ATP/ADP, favoring the hydrolysis of ATP and the release of ADP, and whether these residues cooperate with each other to complete the cycle process.

**Figure 1:**
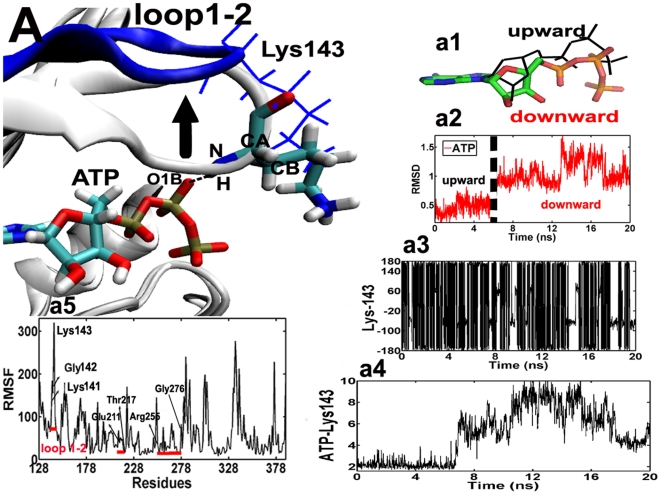
Superposition of the ATP binding sites for the typical snapshots of the Aurora–ATP simulation. 4.35 ns and 8.35 ns. For clarity, only the key region of loop 1–2 is colored in gray (4.35 ns) and blue (8.35 ns). Lys–143 at 4.35 ns is shown in blue line presentation, while residues and ADP at 8.35 ns are shown in stick presentations: cyan for carbon, white for hydrogen, red for oxygen, and blue for nitrogen atoms. As indicated by the black arrow, loop1–2 moves upward, accompanied by the formation and disruption of the hydrogen bond (the dashed line) between Lys–143 (H) and ATP (O1B) in an “open” state. (a1), superposition of ATP focusing on the different conformations of the triphosphate side chain (upward and downward states). (a2), time–dependence of RMSD of ATP. The dashed line depicts the upward and downward conformations of ATP. (a3), time–dependent rotation of Lys–143 about the (CB–CG–CD–CE) dihedral angle. (a4), time evolution of the distance between Lys–143 (H) and ATP (O1B). (a5), time evolution of the RMSF in the Aurora–ATP simulation. The red lines depict residues involved in the ATP binding site: Leu139–Arg–151, Glu–211–Thr–217 and Arg–255–Gly276. The result of the RMSF analysis indicates that in the binary simulation there are significant fluctuations centered around residues 141–143 (loop 1–2).

To explore the key residues to control the cycle of ATP, we have performed a total of 20 ns of all-atom MD simulations of three systems, i.e., Aurora-ATP, Aurora-ADP and Aurora-ADP-TPX2, in explicit water. Fig. 1.a2 shows that the RMSD of ATP inside the binding pocket vs. simulation time in the Aurora-ATP system. Interestingly, the RMSD of ATP remains ∼0.5 Å during the first 5.2 ns, and it reaches up to ∼0.9 Å and even more in the following 14.8 ns. Further analysis reveals that ATP switches between two conformations (upward and downward states) in its binding site, and the main difference between them lies in the state of phosphorous side chain (Fig. 1.a1), raising the question of how Aurora A regulate the conformational switching between the binding (upward) and the release (downward) states for ATP?

The time evolution of the RMS fluctuation (RMSF) in Fig. 1.a5 shows that the binding of ATP leads to the stability of the ATP binding site (RMSF  = 90∼100 Å) with the exception of residues 141–143 (RMSF  = 160∼320 Å) in loop 1–2, indicating that loop 1–2 is extremely flexible. Loop 1–2 can swing up and down (∼2 Å), as indicated by the black arrow in Fig. 1.A. The movement of this loop makes Lys-143 on the top of ATP undergo significant conformational changes in the entire simulation time as evidenced by its large rotation of angle (CB-CG-CD-CE) from −180°∼180° (Fig. 1.a3). In the first 6.0 ns, a stable hydrogen bond (H-bond) is formed between Lys-143 (H) and ATP (O1B) with a H-bond length of ∼2.5 Å (Fig. 1.a4). However after 6.0 ns, the interaction between them is suddenly broken, resulting in a significant increase of RMSF from 27 to 320 Å, and this residue freely flips up and downward around the vertical plane for the loop 1–2 over 12 ns (Fig. 1.A). Interestingly, at the last 2 ns, Lys-143 rotates downward again (30°) and is weakly H-bounded to ATP (O1B) with the H-bond length of 3.5±0.5 Å (Fig. 1.a4). From this process, we can find that Lys-143 vividly captures the small molecule ATP in an intermittent manner, which is distinct from its motion in the Aurora-ADP-TPX2 system (Fig. 1. A). This formation and disruption of H-bond explains why the phosphorous side chain of ATP flip up (H-bond formed) at the beginning but downward (H-bond broken) in the end. The upward conformation might indicate the intimate binding of ATP in the pocket, yet the downward mode might indicate a release trend of phosphate group from the protein. We thus conclude that Lys-143 constitutes a critical switch governing the binding or even release of ATP in the binding site.

**Figure 2:**
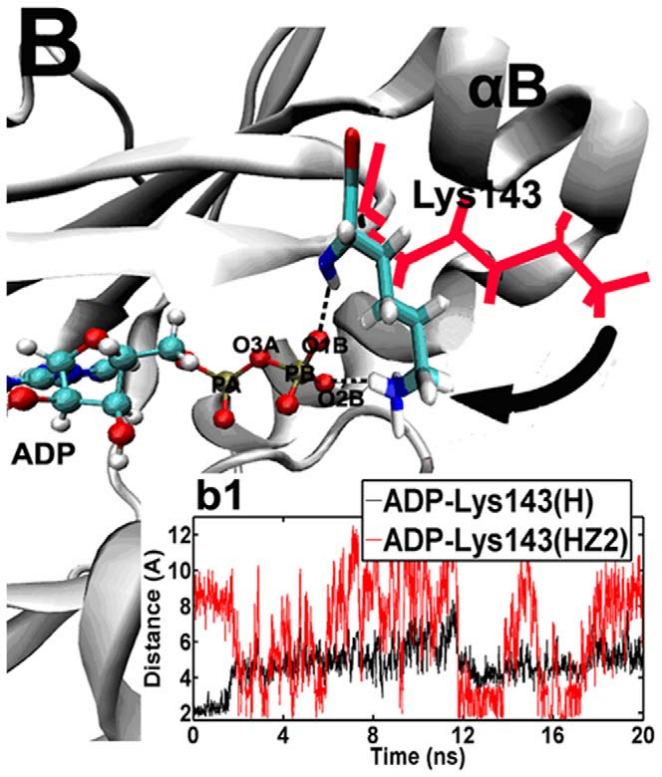
Superposition of the ATP binding sites for the typical snapshots of the Aurora–ADP simulation. 1.0 ns and 5.0 ns. For clarity, the key residue Lys–143 is shown in red line at 1.0 ns, while in sticks at 5.0 ns (cyan, carbon atoms; white, hydrogen atoms; blue, nitrogen atoms and red, oxygen atoms). After the first 1.6 ns, the hydrogen bond between Lys–143 (H) and ATP (O1B) is broken, but instead, the amino side chain of Lys–143 (HZ2) rotates to be contiguous to ATP, thus forming a new hydrogen bond (the dashed line) with the small molecular (O2B) in a “closed” state. The movement of Lys–143 is indicated by using the arrow. (b1), time evolutions of the distance between ADP (O1B) and Lys–143 (H and HZ2) in the Aurora–ADP simulation.

After ATP hydrolysis, Lys-143 further rotates into the ATP binding site and exhibits a “closed” conformation. (Fig. 2. B). Interestingly, in the first 1.6 ns, the backbone hydrogen of Lys-143 (H) generates an H-bond (H-1) with ADP (O1B) (Fig. 2. b1). However, in the subsequent time, the amino group of this residue undergoes a dramatic 60° clockwise rotation (Fig. 2. B), resulting in the disruption of the old H-1 and the simultaneous formation of a new H-bond (H-2) between the side chain of Lys-143 (HZ2) and ADP (O1B) (Fig. 2. b1). This finding suggests that Lys-143 can successfully “hook” the β-phosphate of ADP in this “closed” state (Fig. 2.B). However, Lys-143 still rotates up- and downward remarkably (35°) in the following 18.4 ns, leading to intermittent formation of H-2 in the Aurora-ADP complex (Fig. 2. b1). This result might imply that Lys-143 acts as a potential driving force to pull this ADP out of the binding pocket. As ADP release is the last step to complete kinase activity [20], we thus suggest that the conformational change of Lys-143 plays an important role in this dissociation process.

**Figure 3:**
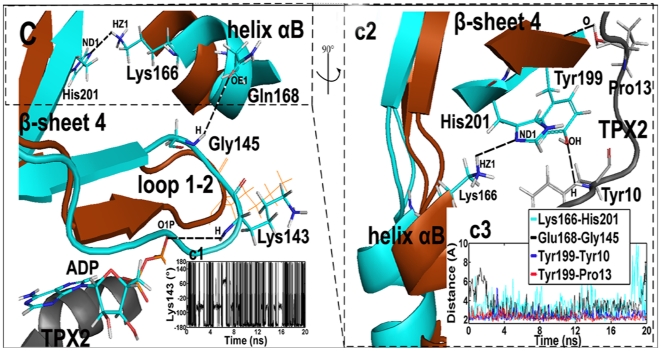
Superposition of the average structures of the Aurora–ADP (brown) and the Aurora–ADP–TPX2 (cyan) simulations. Residues in the binary structure are shown in brown line presentation, while residues in the ternary complex and ADP are shown in stick presentations: cyan for carbon, white for hydrogen, red for oxygen, and blue for nitrogen atoms. The TPX2 fragment is displayed in the gray ribbon. The position of the binding region among helix αB, β–sheet 4 and the upstream of TPX2 is circled in dashed lines. After TPX2 binding, Tyr–199 respectively forms a H–bonds with Tyr–10^TPX2^ (H) and Pro–13^TPX2^(O) (the dashed lines) to pull β–sheet 4 to be more contiguous to helix αB. Due to the decreased distance between β–sheet 4 and helix αB, His–201 (ND1) interacts with Lys–166 (HZ1) to force helix αB to undergo a rotation movement (∼1 Å). This process causes the formation of a H–bond between Gln–168 (OE1) in helix αB and Gly–145 (H) in loop 1–2. As a result, the constrained loop 1–2 leads to a stable, “open” state of Lys–143. (c1), time–dependent rotation of Lys–143 about the (CG–CB–CA–N) dihedral angle in the ternary simulation. (c2), the view is rotated 90° about the y axis relative to (C). (c3), time evolutions of the distance between Lys–166 (HZ1) and His–201 (ND1), Gln–168 and Gly–145 (H), Tyr–199 (OH) and Tyr–10^TPX2^ (H), and Tyr–199 (H) and Pro–13^TPX2^ (O) in the ternary simulation.

In a contrast with the “closed” state for Lys-143 (switch-1), TPX2 binding makes this residue project out of the ATP binding site, in an “open” conformation (see Movie S1). In the ternary system, the binding of TPX2 initially forces loop 1–2 to undergo an upward movement (∼1 Å) and open the ATP binding site, causing Lys143 to rotate with a large fluctuation from 180° to −180°. However, switch-1 still maintains its stable “open” configuration (<−100°) for about 60% of the simulation time (see its angle (CG-CB-CA-N) in Fig. 3.c1). In this case, Lys-143 does not form a H-bond with ADP as evidenced by the distance (∼4 Å) between them (data not shown), showing that ADP becomes more free when Lys-143 opens the ATP binding site. Further, compared with Aurora-ADP system, we observe that triggered by the binding of TPX2, the conformational state of Lys-143 is shifted more stable in the ternary system, since RMSF of Lys-143 is 67.24 Å in the ternary complex, whereas is 144.76 in the binary complex. Such a transition shows a potential cooperation of Lys-143 and ADP, i.e., Lys-143 can swing ADP and favor its release from the binding pocket.

Further analysis shows a H-bond network is responsible for the stable open-state of Lys-143, which is produced between TPX2 and β-sheet 4 close to loop 1-2 and helix αB, including H-bonds of Tyr-199 (OH) and Tyr-10^TPX2^ (H), Tyr-199 (H) and Pro-13^TPX2^ (O) (Fig. 3.c2). The binding of TPX2 forces β-sheet 4 to undergo a translation movement to ATP direction (∼0.5 Å), definitely reducing the space between helix αB and β-sheet 4 (Fig. 3.C), which results in the formation of H-bond between His-201 (ND1) and Lys-166 (HZ1) (Fig. 3.c2). In this process, helix αB rotates clockwise (∼25°) and approaches more contiguous to loop 1-2 (∼1 Å). As a result, Gln-168 (OE1) in helix αB forms a H-bond with Gly-145 (H) in loop 1–2 as evidenced by the H-bond distance (∼3.5 Å) (Fig. 3. c3). This H-bond interaction exerts a constraint force on loop 1-2, thereby leading to conformational stable Lys-143. This analysis thus suggests that the stability of secondary structure in the loop 1–2 that facilitates the swing and open conformation of Lys-143 is regulated by a considerable concomitant consolidation of the TPX2 fragment.

### 2 Switch-2 in the activation loop

Aurora A kinase activity depends on autophosphorylation of Thr-288 in the activation loop. TPX2 binding locks the catalytic domain of the kinase into an active configuration to keep the phosphorylated Thr-288 into a phosphatase-inaccessible conformation [21]. The X-ray crystal structure has released that the binding site of TPX2 is more than 20 Å away from P·Thr288, implying that Aurora A should exhibit certain conformational transition in its N-terminal lobe upon binding of TPX2, and such a global alteration in kinase conformation might be associated with certain allosteric characteristics of the activation loop where Thr288 is located. However, how does the binding of TPX2 influence P·Thr288? Whether there exist certain actions from helix αC, which regulates P·Thr288 in an animate (inward) or catalyzed (outward) state, since the downstream stretch (residues 26–43) of TPX2 directly interacts with the helix αC of Aurora A [16]?

To address these questions, we compare the motion of the activation loop and its vicinity over the 20 ns trajectories of Aurora-ADP and Aurora-ADP-TPX2. The RMSF analysis has shown that the flexibility of Arg-180 in helix αC significantly differs in the binary and ternary simulations (94.19 Å for Aurora-ADP, and 9.28 Å for Aurora-ADP-TPX2), which may indicate that this residue is functionally associated with Aurora A activity.

**Figure 4:**
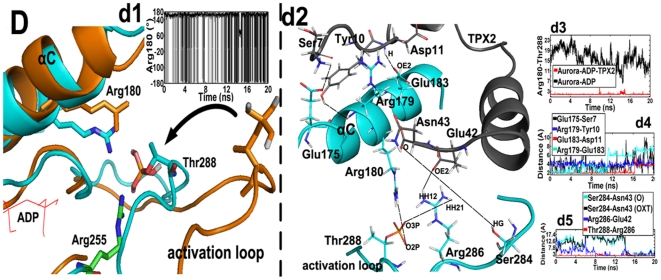
Superposition of the average structures of the Aurora–ADP (brown) and the Aurora–ADP–TPX2 (cyan) systems. Residues in the binary structure are shown in brown stick presentation, while residues in the ternary complex are shown in colored stick presentations: cyan for carbon, white for hydrogen, red for oxygen, and blue for nitrogen atoms. ADP is displayed in the red line. Upon the binding of TPX2, Arg–180 (HH12) forms a hydrogen bond with P·Thr–288 (O2P) to pull the latter residue in an inward conformation. The movement of P·Thr–288 is indicated by using an arrow. (d1), time–dependent rotation of Arg–180 about the (C–CA–CB–CG) dihedral angle in the binary simulation. (d2), side view of the binding region between helix αC and the upstream of TPX2. The Aurora A and the TPX2 fragment are respectively colored cyan and gray. The first H–bond network is formed between Glu–175 (OE2) and Ser–7^TPX2^ (H3), Arg–179 (H) and Tyr–10^TPX2^ (OH), Glu–183 (OE2) and Asp–11^TPX2^ (H), and Arg–179 (HH21) and Glu–183 (OE2) (the dashed lines), thus constraining the fluctuation of Arg–180. The second hydrogen bond network is formed between Ser–284 (HG) and Asn–43^TPX2^ (O), Ser–284 (HG) and Asn–43^TPX2^ (OXT), Arg–286 (HH12) and Glu–42^TPX2^ (OE2), and Thr–288 (O3P) and Arg–286 (HH21) (the dashed lines), thus constraining the fluctuation of Thr–288. (d3), time evolutions of the distance between Arg–180 and P·Thr–288 in the binary and the ternary simulations. (d4), time evolutions of the distance between Glu–175 and Ser–7^TPX2^, Arg–179 and Tyr–10^TPX2^, Glu–183 and Asp–11^TPX2^, Arg–179 and Glu–183, and Arg–251 and Glu–37^ TPX2^ in the ternary simulation. (d5). time evolutions of the distance between Ser–284 and Asn–43^TPX2^, Ser–284 and Asn–43^TPX2^, Arg–286 and Glu–42^TPX2^, and Thr–288 and Arg–286 in the ternary simulation.

In the binary system, the free side chain of Arg-180 dramatically and transiently oscillates from −180° to 180° as evidenced by its dihedral angle (C-CA-CB-CG), and stays at 180° in most of the simulated time (Fig. 4.d1). On this time scale, Arg-180 exhibits a relatively stable conformation (180°) and protrudes outside the ATP binding site in an “open” state (Fig. 4.D). In contrast to the large fluctuation of Arg-180, the activation segment only undergoes slightly changes (RMSF = 35 Å). The slight in-outward movement of the activation segment leads to small-scale motions of the phosphate group of P·Thr-288 with its varied dihedral angel (O2P-P-OG-CB) between −160° and 170°. Thus this residue can orient it in the outward direction so as to permit the accession of phosphatase such as PP1, with a consequence of loss of the phosphate group from this amino acid [19]. Given the “open” states of both Arg-180 and P·Thr-288 in the binary complex, clearly no H-bond is formed between them as their inter-residue distance is ∼12 Å (Fig. 4.d4). However, once TPX2 binds to Aurora A, Arg-180 rotates downward (40°) and becomes a rigid, “closed” state (RMSF  = 9.28 Å) (Fig. 4.D). Meanwhile, the activation loop also significantly rotates inward to ATP (3 Å), thus maintaining P·Thr-288 in an active conformation in the ternary complex (Fig. 4.D). In this “closed” state, a stable H-bond is formed between Arg-180 and P·Thr-288 and the H-bond length keeps 2.1 Å in Fig. 4.d4.

We further find that the conformational transitions of Arg-180 and P·Thr-288 are respectively induced by two independent H-bond networks, i.e., 1) the upstream of TPX2 vs Helix αC (network-1); 2) the down stream of TPX2 vs activation loop (network-2). Network-1 includes H-bonds of Glu-175 (OE2) and Ser-7^TPX2^ (H3), Arg-179 (H) and Tyr-10^TPX2^ (OH), Glu-183 (OE2) and Asp-11^TPX2^ (H), and Arg-179 (HH21) and Glu-183 (OE2) (Fig. 4.d3). This network strengthens the contact between TPX2 and helix αC, which stabilizes this helix and also forces it to slightly descend by 0.3 Å, thereby causing a strong H-bond (2 Å) between Arg-180 and P·Thr-288 (Fig. 4. d3). And network-2 is composed of Ser-284 (HG) and Asn-43^TPX2^ (O), Ser-284 (HG) and Asn-43^TPX2^ (OXT), Arg-286 (HH12) and Glu-42^TPX2^ (OE2), as well as Thr-288 (O3P) and Arg-286 (HH21) (Fig. 4.d4). The network-2 makes the activation loop to rotate in a buried conformation as indicated by the black arrow in Fig. 4.D. The motion of this loop subsequently causes P·Thr-288 to flip inward by 100° from its outward state. Combined, the two networks triggered by the binding of TPX2 favors the inward flip of P·Thr-288.

### 3 Cross-correlation of the two switches

As the residues involved in the switch-1 (Lys-143) and switch-2 (Arg-180) are structurally neighboring, we suppose that they might also functionally cooperate with each other. The dynamic cross-correlation map (DCCM) is then used to further investigate the correlations of switch-1 and switch-2. DCCM describes the global correlated motions among residues from the highly anticorrelated (blue) to the highly correlated (red).

**Figure 5:**
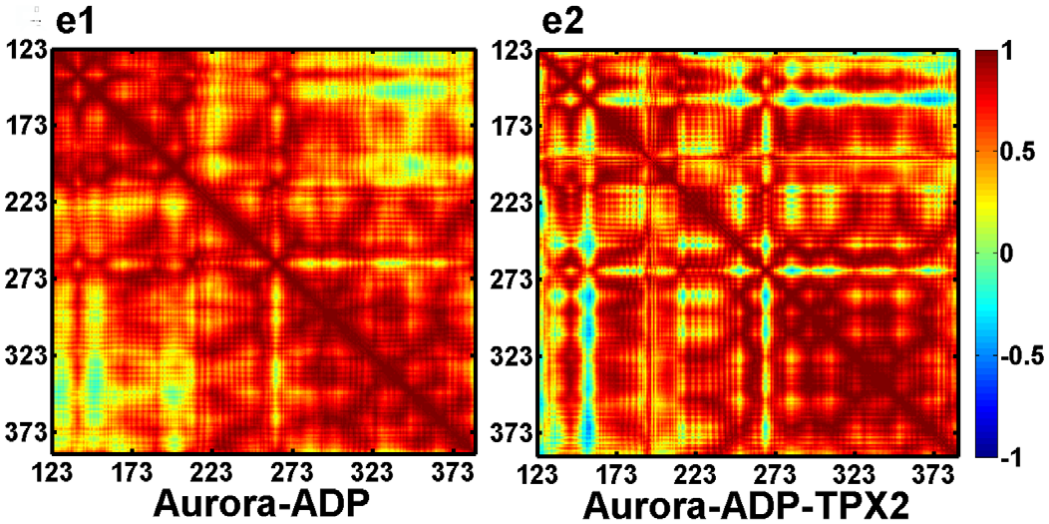
Cross correlation matrices of fluctuations of Aurora A atoms from their average values during the last 10 ns of the binary (e1) and ternary (e2) simulations.

Fig. 5 shows that the fluctuations of Lys-143 and ADP are, on the average, positively correlated in both the binary and ternary systems. Interestingly, the TXP2 binding does not increase the correlation between the two residues with the correlation coefficient (*R* = 0.9) same as the binary simulation of 0.9. Similarly, strong positive correlations are also observed between Arg-180 and P·Thr-288 in the two systems, however, the *R* arises to 0.97 (ternary) from 0.83 (binary) due to TXP2 binding. In addition, we also find strong correlations between switch-1 and switch-2, with a correlation of *R = *0.74 in the binary system, while 0.82 in the ternary system, meaning that inter-switch cooperation is also enhanced upon TPX2 binding.

### 4 Mutations of switch-1 and switch-2

To further map the functionality of the two switches, an additional MD simulation on K143A and R180A mutants is performed. Comparing K143A with the wild type Aurora A, the hydrogen bond interaction between the β-phosphate group of ADP and the backbone amino group of K143A is formed and lasts 1.5 ns due to the increased side chain fluctuation of K143A (RMSF is 158.44 Å for the mutant protein, and is 67.24 for the wild type protein). The formation of the hydrogen bond between ADP and K143A delays the release of ATP, resulting in the interruption of normal ATP cycle. Therefore, the evidence from the mutant ternary system demonstrates the function of swich 1 from another respect.

While for the mutation of Arg-180 to Ala in Aurora A, this mutation actually interrupts the hydrogen bond interaction between Arg-180 and P·Thr-288 in the wild-type Aurora A, leading to the outward flip of P·Thr-288 in the activation loop (see Fig. S2). This is evidenced by the time evolution of the distance (10 Å) between the phosphate group of Thr-288 and the backbone hydrogen of R180A, resulting in the stable open state of P·Thr-288 (Fig. S2). We thus reason that mutation of the two residues to other amino acids with short side chains might respectively prevent the normal cycle of ATP and phosphorylation of Aurora A, therefore blocking activation of the protein.

### Conclusions

The kinase Aurora A is overexpressed in many tumors, and has become an effective target for anticancer drug. There are two routes to inhibiting its function: 1) interfere with its ability to interact with its binding partners, such as TPX2; 2) occupy the catalytic ATP binding site. Targeting protein–protein interactions with small-molecule inhibitors and selectively targeting the enzymatic activity of kinase with small molecules have proved to be great challenges since the key residues involved in the regulating of ATP cycle and TPX2 binding are still not unveiled. In this study, we present two functional correlated TPX2-dependent switches important for Aurora A activation, which have not been investigated so far. Swith-1 composed of Lys-143 plays an essential role in kinetic cycle of ATP binding, nucleotide hydrolysis, and release of ADP, thus regulating the activity of Aurora A. Switch-2 includes Arg-180, the open and closed states of which restricts the conformational movement of P·Thr-288, thus completing the cell cycle-dependent feedback of phosphorylation/dephosphorylation of Aurora A. Our in-depth study have uncovered that the molecular basis of Aurora A activation is largely determined by the two novel switches, which is important in designing the compounds for treating tumors.

## Methods

### 1 Structural preparation

Initial coordinate of Aurora A was taken from the human X-ray crystal structure [15] (PDB entries 1ol5, 1ol6 and 1ol7). Molecular dynamics (MD) simulations in explicit water have been independently carried out on a simulation time scale of 20 ns for (1) the unbound apo form of the Aurora A in solution (apo-Aurora), (2) Aurora A complexes with the natural substrate ATP (Aurora-ATP), (3) Aurora A complexes with the substrate ADP (Aurora-ADP), (4) Aurora A-ADP complexes with the coactivator TPX2 (Aurora-ADP-TPX2) and (5) Aurora-ADP-TPX2 with the K143A and R180A mutants. The missing residues (Leu22-Gln29 of the TPX2 segment (1ol5), and Gly142, Gly173 and Val174 of Aurora A (1ol6)) were repaired by using the loop search method in the Swiss-PdbViewer (aka DeepView, http://spdbv.vital-it.ch/). After removal of the phosphate group from the phosphorylated Thr287, the crystal structure of Aurora A in complex with ADP and TPX2 (1ol5), was used directly in the simulation of Aurora-ADP-TPX2 complex, and was also applied to generate the starting structure of the Lys143-Ala and Arg180-Ala mutants. This Ala was selected for the reason that no H-bond could be formed in the mutants for this residue. By analyzing the Lys143Ala and Arg180Ala trajectory, we observed that the mutated Aurora A could retain the basic native structure. Similarly, the simulations of Aurora-ATP and Aurora-ADP complexes were respectively generated from the crystal structures 1ol6 and 1ol7. In the absence of crystal structure of apo-Aurora, this apo simulation was also generated from the crystal structure of Aurora A in complex with ADP and TPX2 (1ol5) by removing the ligand and TPX2 from the active site and the phosphate groups of phosphorylated Thr287 and Thr288.

The Amber03 force field [22] was used with xLeap in Amber10 [23] for system setup, and AMBER force parameters for ATP, ADP [24] and Thr with unprotonated phosphate group [25] were derived from AMBER parameter database (www.pharmacy.manchester.ac.uk/bryce/amber). A box of TIP3P water molecules [26] that extended at least 12.0 Å outside the protein was added to solvate to each system (74.98 Å×90.58 Å×90.20 Å for apo-Aurora, 73.10 Å×68.40 Å×75.76 Å for Aurora-ATP, 78.38 Å×75.31 Å×73.09 Å for ADP-Aurora, 70.09 Å×86.58 Å×90.20 Å for Aurora- ADP-TPX2, and 73.60 Å×73.96 Å×69.47 Å for mutant Aurora-ADP-TPX2). To neutralize the system, Na^+^ and Cl^−^ counter ions were added. Ions were placed on the basis of the electrostatic energy of the water oxygens. Finally, the five systems were minimized respectively, usually for 5000 steepest-descent steps followed by 5000 conjugate-gradient steps.

### 2 Simulation Protocol

All simulations were run by the sander module in Amber 10 [25]. MD simulations started with heating the system from 0 to 300 K in 100 ps followed by a 50 ps pressure-constant period to raise the density while still keeping the complex atoms constrained, after which 0.5 ns of equilibration was conducted. The following 20 ns were considered as further equilibration time and analysis was done on the following parts of trajectories. Finally, the production phase was run for 20 ns, considering that each trajectory is long enough to ensure sufficient sampling of the protein's configuration space and the ligand, i.e., allowing the systems to cross the barrier between folded and misfolded free energy basins more than once. MD simulations were run in the NPT ensemble, using periodic boundary conditions, a 2 fs timestep, and SHAKE algorithm [27] to constrain all bonds to hydrogens. The temperature was kept at 300 K using Langevin dynamics [28] with a collision frequency of 1 ps^−1^. The cutoff distance was kept to 8 Å to compute the nonbonded interactions. All the simulations were performed under periodic boundary conditions, and long-range electrostatics was treated by using the particle-mesh-Ewald (PME) method [29]. Snapshots were collected from the stable structures during the last 10 ns of the four simulations for analysis (1snapshot/10 ps).

## Supporting Information

Figure S1 **Ribbon diagram of the Aurora A structure (cyan) oriented to show the relative positions of the C- and N-terminal lobes.** TPX2 is colored in red ribbon, and ADP and Thr-288 are shown in blue sticks.
(tif)

Figure S2 **The average structure of the mutant Aurora-ADP-TPX2 (cyan) simulation.** Residues and ADP in the ternary structure are shown in stick presentation: cyan for carbon, white for hydrogen, red for oxygen, and blue for nitrogen atoms. TPX2 is displayed in the gray ribbon. The dashed lines show the distance between K143A (H) and ADP (O1B), and between R180A (H) and Thr288 (O2P). (a) the time evolution of distance between K143A (H) and ADP (O1B), and between R180A (H) and Thr288 (O2P) in the ternary simulation.
(tif)

Movie S1 **The open and closed motions of switch-1 (Lys-143) and switch-2 (Arg-180) in the Aurora A-ATP, Aurora A-ADP, and Aurora A-ADP-TPX2 simulations.** Aurora A is shown in gray ribbon, Lys-143 and ATP/ADP are shown in stick representation, and Arg-180 and Thr-288 are shown in ball and line presentation.
(AVI)

## References

[1] Fu J, Bian M, Jiang Q, Zhang C (2007). Roles of Aurora Kinases in Mitosis and Tumorigenesis.. Mol Cancer Res.

[2] Marumoto T, Honda S, Hara T, Nitta M, Hirota T (2003). Aurora-A kinase maintains the fidelity of early and late mitotic events in HeLa cells.. J Biol Chem.

[3] Vader G, Lens S (2008). The Aurora kinase family in cell division and cancer.. Biochim Biophys Acta BBA.

[4] Gautschi O, Heighway J, Mack PC, Purnell PR, Lara PN (2008). Aurora Kinases as Anticancer Drug Targets.. Clin Cancer Res.

[5] Zhou H, Kuang J, Zhong L, Kuo WL, Gray J (1998). Tumour amplified kinase STK15/BTAK induces centrosome amplification, aneuploidy and transformation.. Nat Genet.

[6] Giet R, Petretti C, Prigent C (2005). Aurora kinases, aneuploidy and cancer, a coincidence or a real link?. Trends Cell Biol.

[7] Marumoto T, Zhang D, Saya H (2005). Aurora-A–A guardian of poles.. Nat Rev Cancer.

[8] Moyer ML, Gilbert SP, Johnson KA (1998). Pathway of ATP hydrolysis by monomeric and dimeric kinesin.. Biochemistry.

[9] Goodsell DS, Cassier P (1996). Our molecular nature: The body's motors, machines and messages..

[10] Hirota T, Kunitoku N, Sasayama T, Marumoto T, Zhang D (2003). Aurora-A and an interacting activator, the LIM protein Ajuba, are required for mitotic commitment in human cells.. Cell.

[11] Satinover DL, Leach CA, Stukenberg PT, Brautigan DL (2004). Activation of Aurora–A kinase by protein phosphatase inhibitor–2, a bifunctional signaling protein.. Proc Natl Acad Sci USA.

[12] Pugacheva EN, Golemis EA (2005). The focal adhesion scaffolding protein HEF1 regulates activation of the Aurora-A and Nek2 kinases at the centrosome.. Nat Cell Biol.

[13] Hutterer A, Berdnik D, Wirtz–Peitz F, Zigman M, Schleiffer A (2006). Mitotic activation of the kinase Aurora-A requires its binding partner Bora.. Dev Cell.

[14] Bibby RA, Tang C, Faisal A, Drosopoulos K, Lubbe S (2009). A cancer–associated Aurora A mutant is mislocalized and misregulated due to loss of interaction with TPX2.. J Biol Chem.

[15] Bayliss R, Sardon T, Vernos I, Conti E (2003). Structural basis of Aurora-A activation by TPX2 at the mitotic spindle.. Mol Cell.

[16] Zhao B, Smallwood A, Yang J, Koretke K, Nurse K (2008). Modulation of kinase-inhibitor interactions by auxiliary protein binding: Crystallography studies on Aurora A interactions with VX–680 and with TPX2.. Protein Sci.

[17] Agnese V, Bazan V, Fiorentino FP, Fanale D, Badalamenti G (2007). The role of Aurora-A inhibitors in cancer therapy.. Ann Oncol.

[18] Nowakowski J, Cronin CN, McRee DE, Knuth MW, Nelson CG (2002). Structures of the cancer-related Aurora-A, FAK, and EphA2 protein kinases from nanovolume crystallography.. Structure.

[19] Groen AC, Needleman D, Brangwynne C, Gradinaru C, Fowler B (2008). A novel small-molecule inhibitor reveals a possible role of kinesin–5 in anastral spindle–pole assembly.. J Cell Sci.

[20] Lu B, Wong CF, McCammon JA (2005). Release of ADP from the catalytic subunit of protein kinase A: A molecular dynamics simulation study.. Protein Sci.

[21] Eyers PA, Maller JL (2003). Regulation of Xenopus Aurora A activation by TPX2.. J Bio Chem.

[22] Wang J, Cieplak P, Kollman PA (2000). How well does a restrained electrostatic potential (RESP) model perform in calculating conformational energies of organic and biological molecules.. J Comput Chem.

[23] Case DA, Darden TA, Cheatham TE, Simmerling CL, Wang J (2008).

[24] Meagher KL, Redman LT, Carlson HA (2003). Development of polyphosphate parameters for use with the AMBER force field.. J Comput Chem.

[25] Homeyer N, Horn AHC, Lanig H, Sticht H (2006). AMBER force field parameters for phosphorylated amino acids in different protonation states: phosphoserine, phosphothreonine, phosphotyrosine and phosphohistidine.. J Mol Model.

[26] Jorgensen WL, Chandrasekhar J, Madura JD, Impey RW, Klein ML (1983). Comparison of simple potential functions for simulating liquid water.. J Chem Phys.

[27] Ryckaert JP, Ciccotti G, Berendsen HJC (1977). Numerical integration of the Cartesian equations of motion of a system with constraints: molecular dynamics of n–alkanes.. J Comput Phys.

[28] Wu X, Brooks BR (2003). Self–guided Langevin dynamics simulation method.. Chem Phys Lett.

[29] Darden T, York D, Pederson L (1993). Particle mesh Ewald: An N log (N) method for Ewald sums in large systems.. J Chem Phys.

