# Supplementary figures and images for: Two TPX2-Dependent Switches Control the Activity of Aurora A
Source: PLoS One. 2011 Feb 9;6(2):e16757. doi: 10.1371/journal.pone.0016757 (PMC3036663; doi:10.1371/journal.pone.0016757)

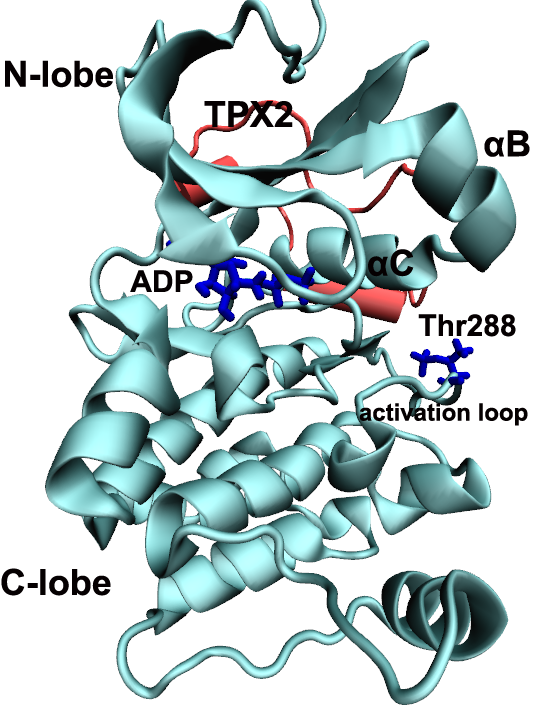

Supplement: Figure S1 — Ribbon diagram of the Aurora A structure (cyan) oriented to show the relative positions of the C- and N-terminal lobes. TPX2 is colored in red ribbon, and ADP and Thr-288 are shown in blue sticks. (tif) [file pone.0016757.s001.tif]

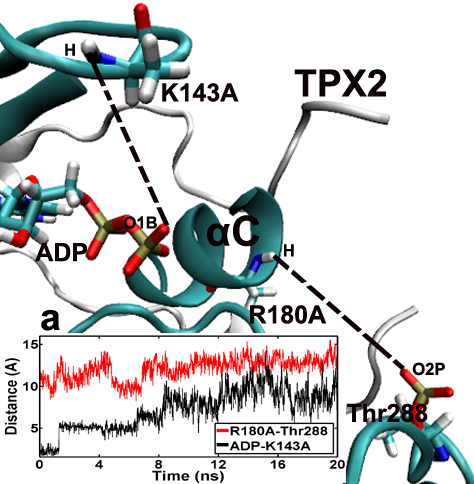

Supplement: Figure S2 — The average structure of the mutant Aurora-ADP-TPX2 (cyan) simulation. Residues and ADP in the ternary structure are shown in stick presentation: cyan for carbon, white for hydrogen, red for oxygen, and blue for nitrogen atoms. TPX2 is displayed in the gray ribbon. The dashed lines show the distance between K143A (H) and ADP (O1B), and between R180A (H) and Thr288 (O2P). (a) the time evolution of distance between K143A (H) and ADP (O1B), and between R180A (H) and Thr288 (O2P) in the ternary simulation. (tif) [file pone.0016757.s002.tif]
